# Targeting CD47-SIRPa axis shows potent preclinical anti-tumor activity as monotherapy and synergizes with PARP inhibition

**DOI:** 10.1038/s41698-023-00418-4

**Published:** 2023-07-19

**Authors:** Hussein Al-Sudani, Ying Ni, Philip Jones, Huseyin Karakilic, Lei Cui, Lisa D. S. Johnson, Peter G. Rose, Alexander Olawaiye, Robert P. Edwards, Robert A. Uger, Gloria H. Y. Lin, Haider Mahdi

**Affiliations:** 1 Internal Medicine Department, Einstein Medical Center Montgomery, Philadelphia, PA USA; 2 Center for Immunotherapy & Precision Immuno-Oncology, Lerner Research Institute, Cleveland Clinic, 9500 Euclid Avenue, Cleveland, OH 44195 USA; 3 Magee Women’s Research Institute, University of Pittsburgh, Pittsburgh, PA 15213 USA; 4 Trillium Therapeutics Inc, 2488 Dunwin Dr., Mississauga, ON L5L 1J9 Canada; 5 Section of Gynecologic Oncology, Women’s Health Institute, Cleveland Clinic, 9500 Euclid Avenue, Cleveland, OH USA; 6 Magee Women’s Hospital, University of Pittsburgh Medical Center, Pittsburgh, PA 15213 USA; 7 Hillman Cancer Center, University of Pittsburgh, Pittsburgh, PA 15213 USA

**Keywords:** Cancer genomics, Drug development, Ovarian cancer

## Abstract

The objective was to correlate CD47 gene expression with resistance to immune checkpoint inhibitors (ICI) in tumor tissue of gynecological cancer (GC). Further, we sought to assess the efficacy of targeting CD47 pathway alone and in combination in pre-clinical ovarian cancer (OC) models. We performed transcriptomic analyses in GC treated with ICI. Signaling pathway enrichment analysis was performed using Ingenuity Pathway Analysis. Immune cell abundance was estimated. CD47 expression was correlated with other pathways, objective response, and progression-free survival (PFS). Anti-tumor efficacy of anti-CD47 therapy alone and in combination was investigated both in-vitro and in-vivo using cell-line derived xenograft (CDX) and patient-derived xenograft (PDX) models. High CD47 expression associated with lower response to ICI and trended toward lower PFS in GC patients. Higher CD47 associated negatively with PDL1 and CTLA4 expression, as well as cytotoxic T-cells and dendritic cells but positively with TGF-β, BRD4 and CXCR4/CXCL12 expression. Anti-CD47 significantly enhanced macrophage-mediated phagocytosis of OC cells in-vitro and exhibited potent anti-tumor activity in-vivo in OC CDX and PDX models. In-vitro treatment with PARPi increased CD47 expression. Anti-CD47 led to significantly enhanced in-vitro phagocytosis, enhanced STING pathway and synergized in-vivo when combined with PARP inhibitors in *BRCA*-deficient OC models. This study provides insight on the potential role of CD47 in mediating immunotherapy resistance and its association with higher TGF-β, BRD4 and CXCR4/CXCL12 expression. Anti-CD47 showed potent anti-tumor activity and synergized with PARPi in OC models. These data support clinical development of anti-CD47 therapy with PARPi in OC.

## Introduction

There is considerable interest in the potential role of immunotherapy in ovarian cancer (OC) and other gynecologic cancers. The impact of immune checkpoint inhibitors (ICI) has been significant, with durable response rates in several cancers. However, the response rates for ICI in OC are low, ranging from 11–15% in platinum-resistant, recurrent setting^1,2^. Therefore, it is critical to explore alternative strategies to target immunosuppressive factors with the tumor immune microenvironment.

Tumor associated macrophages (TAMs) in ovarian cancer have been shown to support tumor growth by promoting angiogenesis and suppressing the immune response. TAMs are the predominant immune cells in the ovarian cancer tumor microenvironment^3^. The immune suppressive T-regulatory cell (T-reg) function is supported by TGF-β. Mechanistic interactions between T-regs and TAMs have been reported^4^. Prior studies showed that TAMs modulate tumor expression of VEGF-A, MMP-9, and ARG1, promoting a pro-angiogenic and immunosuppressive environment. In fact, TAM and T-reg abundance is predictive of outcome and therapy resistance in ovarian cancer^3,5,6^*.* Therefore, it is attractive to consider strategies that target myeloid cells to switch the TAM phenotype from immunosuppressive to pro-inflammatory, which might alter the tumor immune microenvironment in ovarian cancer.

CD47 is a transmembrane protein that is highly expressed on the surface of many solid tumors. It is a macrophage immune checkpoint and provides a “don’t eat me” anti-phagocytic signal by interacting with the ligand signal regulatory protein α (SIRP-α) on macrophages, leading to inhibition of phagocytosis and preventing engulfment of the tumor cells by immune cells^7–9^. *CD47* mRNA expression has been shown to predict survival in several solid tumors^3,7^. Anti-CD47 antibodies have been shown to enable phagocytosis by macrophages, induce phenotype switch from M2 to M1 TAMs and inhibit or eliminate tumor growth^7,10^. In fact, anti-CD47 therapy was shown to target and eliminate cancer stem cells in several solid tumor models^7,10^.

In our prior study, immune cells profiling of a large cohort of patients (*n* = 719) demonstrated a predominant myeloid gene signature. The cohort included patients diagnosed with ovarian cancer (*n* = 376) and endometrial cancer (*n* = 305) from TCGA^3^, the abundance of immune cell subsets was determined as in Becht et al. ^11^. Further in the same study, we showed that higher *CD47*, *TGF-β* expression and myeloid cells infiltrate predicted lower survival outcome^11–13^.

CD47 blockade synergizes with cytotoxic therapies that stress tumor cells and induce immunogenic cell death. The damage associated molecular patterns (DAMP) cell damage signals, especially calreticulin, are induced by immunogenic cell death and expressed on the cell surface leading to enhanced phagocytic activity of the macrophages (“eat me signal”). Activation of dendritic cells subsequently stimulates the activity of cytotoxic T-cells and NK cells. This effect is enhanced when CD47 (“don’t eat me signal”) is blocked. Anti-CD47 has been shown to synergize with several chemotherapeutic agents including cisplatin, doxorubicin, paclitaxel, and gemcitabine. In fact, chemotherapy has been shown to induce up-regulation of CD47 and PDL1 expression on tumor cells^14–16^. However, it is unknown if PARP inhibition up-regulate CD47 expression or not? and if targeting CD47 will synergize with PARP inhibition.

In this study we sought to investigate role of *CD47* mRNA expression in relation to immunotherapy resistance and correlate its expression with other immune pathways. Further, we investigated the activity of anti-CD47 therapy alone and in combination in preclinical models of ovarian cancer for potential future developmental strategy in combination approach.

## Results

### Immune cells characterize the tumor immune microenvironment and *CD47* expression associated negatively with cytotoxic T-cells and dendritic cells

Our cohort included tumor samples from a retrospective series of 47 patients diagnosed with gynecology cancer (included endometrial, cervical, or ovarian cancers). All patients were treated with the immune checkpoint inhibitors nivolumab, pembrolizumab or avelumab. Of all 47 patients, 20 (42%) had an objective response (either partial or complete response by RECIST 1.1). Baseline patient characteristics and clinical data of our patients are summarized in Supplementary Table 1.

**Fig. 1:**
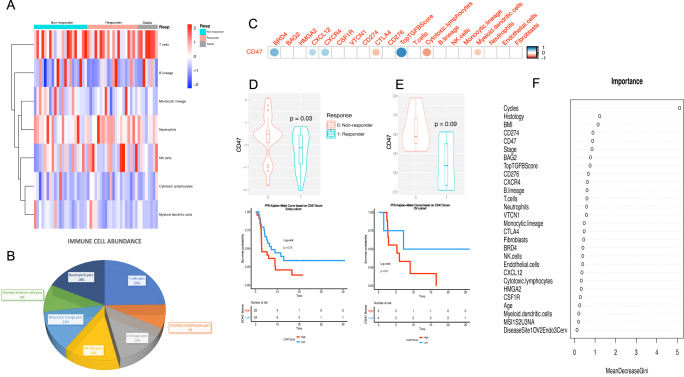
Different immune cell abundance for cohort of 49 patients with gynecologic cancer who received immunotherapy with immune checkpoint inhibitors and correlation of *CD47* with immune checkpoint and other genes involved in immune response and surveillance. **A** Heatmap of estimated immune cell abundance, sorted by responders and non-responders. **B** Pie chart of the prevalence of each immune cell abundance among all samples. **C** Correlation plot between *CD47* and different immune cells as well as other markers including genes involved in immune response and surveillance. The correlation plot was made using “corrplot” R package, with the color of the circles shows the absolute value of corresponding correlation coefficients and area of the circles shows the significance. **D**, **E** *CD47* expression in correlation with objective response to immunotherapy and progression-free survival while on immunotherapy. Box inside the violin plot visualizes summary statistics including the lower and upper hinges correspond to the first and third quartiles (the 25th and 75th percentiles) and center line as the median. D: CD47 RNA expression among responders to immunotherapy compared to those who did not respond in the entire cohort. *CD47* was significantly higher among responders compared to non-responders (*p* = 0.03) (Top). Kaplan–Meier curves of PFS based on *CD47* expression in entire cohort (Bottom). **E** *CD47* RNA expression among responders to immunotherapy compared to those who did not respond in ovarian cancer patients only. *CD47* was higher among responders compared to non-responders (*p* = 0.09) (Top). Kaplan–Meier curves of PFS based on *CD47* expression in ovarian cancer patients (Bottom). *CD47* group “High” or “low” was defined by median RNA expression of *CD47*. **F** The estimated importance of all relevant variables for predicting ICI responses using a Random Forest Model.

Using transcriptome data, we profiled the immune cells that comprise the tumor immune microenvironment. We characterized 7 immune composition subsets (with endothelial and fibroblast cells) in silico, by extracting genes representing these immune cells and activating status using our bulk RNAseq data^11^. We then calculated the cell type signature score based on the representing genes in each functional annotation in Fig. 1A. Based on this bioinformatic estimation, in general, these patients had relatively higher expression of myeloid lineage cells (13%) (consistent with tumor associated macrophage) and neutrophils (18%) compared to lower expression of cytotoxic T-cells (7%) and dendritic cells (9%) (Fig. 1B). *CD47* RNA expression significantly associated with lower cytotoxic T-cells (*p* < 0.001) and dendritic cells (*p* = 0.038) (Fig. 1C).

### *CD47* expression associated with markers indicating immunosuppressive immune environment

Tumor expression of *CD47* RNA was correlated with immune checkpoint molecules like *PDL1* and *CTLA4*. Higher *CD47* expression associated with lower *CTLA4* (*p* = 0.01) and *PDL1* (*p* = 0.05) expression (Fig. 1C). These correspond to the negative association between *CD47* expression and activated cytotoxic T-cells.

We then sought to correlate *CD47* RNA expression with other immune markers known to have an immunosuppressive role in the stroma. Interestingly, *CD47* expression correlated positively with higher *BRD4* (*p* = 0.0002), TGF-β score using our reported 6-gene signature (*p* < 0.0001) as well as *CXCL12* (*p* = 0.024) and *CXCR4* (*p* = 0.004) expressions (Fig. 1C). These data support that *CD47* associates with immunosuppressive factors that promote an immunosuppressive environment favoring macrophage prominent TME and mediated by immunosuppressive stromal factors. Correlative expression between other markers is outlined in Supplementary Fig. 1.

### Higher *CD47* mRNA expression was associated with worse response to ICI immunotherapy

We then correlated clinical outcome data such as objective response rate (ORR) and progression-free survival (PFS) with *CD47* RNA expression. Interestingly, *CD47* expression was significantly higher among those who did not respond to immunotherapy compared to those who responded (*p* = 0.03) (Fig. 1D). Similarly, and despite smaller size, a trend toward lower *CD47* expression was seen among ovarian cancer patients who responded to ICI compared to those who did not respond (*p* = 0.09) (Fig. 1D). A trend toward lower PFS was seen among those with high *CD47* expression compared to lower *CD47* expression both in the entire cohort (Fig. 1D) and ovarian cancer sub-cohort (Fig. 1E). We correlated other genes related to immunosuppressive microenvironment with response to ICI, only *CD47* was negatively associated with response to ICI (Supplementary Fig. 1) in our multivariate analysis when controlled for cytotoxic T-cells and dendritic cells (Supplementary Table 2). These data support association of CD47-SIRPa axis and tumor associated myeloid cells with immunosuppressive tumor immune environment and possibly resistance to ICI.

To validate the association between *CD47* expression and survival we observed in our gynecology cancer patients, we tested *CD47* expression as a biomarker to predict tumor immune response using tumor pre-treatment expression profile database, using the online tool TIDE: Tumor Immune Dysfunction and Exclusion as described in Method. Interestingly, compared to other existing biomarkers, *CD47* showed good predictive value (AUC > 0.7) in 2 studies (Nathanson 2017_CTLA4_Melanoma_Pre and Miao2018_ICB_Kidney_Clear, Supplementary Fig. 2A) and a significant negative association with available overall survival in 2 studies (Liu2019_PD1_Melanoma_Ipi and Braun2020_PD1_Kidney_Clear) (Supplementary Fig. 2). We also investigated differentially expressed genes and pathways between low and high CD47 expression as noted in Supplementary Fig. 3.

### CD47 as the important molecular factor predicting ICI response with random forest model

We then incorporated our molecular markers, clinical features, and demographic variables into a random forest classifier, for its unique advantages in dealing with small sample sizes, high-dimensional feature space, and flexible data structures. The variables included TGF-β score, *CD47*, other immune markers known to have an immunosuppressive role in the stroma: *CXCL12, BAG2, CXCR4, BRD4, HMGA2, CSF1R, CD276, VTCN1*, some previously reported to be associated with ICB response *CD274, CTLA4, VTCN1*, estimated immune cell abundance from RNAseq analysis (*N* = 9), and clinical-pathological features: age, gender, microsatellite instability (MSI) status, body mass index (BMI), disease type as endometrial, ovarian, or cervical cancer, histology type as endometrioid, serous, or squamous, and treatment cycle count (Fig. 1F).

With this model, we quantified the relative importance of the various factors in explaining patient-to-patient response variation (Fig. 1F). When examining single feature contributions to response prediction, treatment cycle had the biggest influence (Fig. 1F), consistent with its known link with response. In addition, the kind of histology and body mass index are also significant in determining the result of IBC treatment. *CD274* (PD-L1) and *CD47* ranked as the two most influential factors leading to the expected response among all molecular markers.

### Anti-CD47 monotherapy enhanced macrophage-mediated in-vitro phagocytosis and inhibited OC tumor growth in xenograft models

**Fig. 2:**
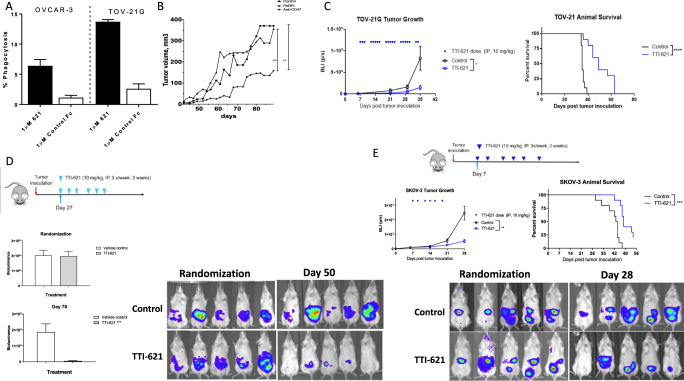
Preclinical in-vitro and in-vivo activity of anti-CD47 therapy in ovarian cancer cells. **A** in-vitro phagocytosis of ovarian cancer cells (OVCAR3 and TOV-21G) with macrophages in the presence of 1uM TTI-621 or control Fc. **B** in-vivo anti-tumor activity of anti-CD47 therapy with Hu-5F9 compared to control and Olaparib in PDX model of high grade serous ovarian cancer (chemotherapy and PARPi resistant model). PDX tumor was implanted subcutaneously, treatment was started when tumor was established. Anti-CD47 was given intraperitoneally twice a week for four weeks. Olaparib was given 50 mg/kg oral gavage 5 times a week till end of the experiment. **C** NOD/SCID mice were implanted (IP) with TOV-21G cells and treated with TTI-621 (IP 10 mg/kg starting day 7 till day 35). Tumor growth measured by BLI. Kaplan-Meier plots of survival. TTI-621 significantly inhibited tumor growth by both BLI (*p* = 0.025) and animal survival (*p* < 0.0001). **D** TTI-621 treatment schedule for NOD/SCID mice implanted intraperitoneally (IP) with luciferase expressing OVCAR-3 cells. Representative bioluminescence images (BLI) of mice at randomization and day 50. Tumor burden measured by BLI at randomization and day 78. TTI-621 significantly inhibited tumor growth (*p* = 0.002). No toxicity was observed for mice treated with TTI-621 as measured by body weight change. **E** NOD/SCID mice were implanted (IP) with SKOV-3 cells and treated with TTI-621. Representative BLI images at randomization (day 6) and day 28. Tumor growth measured by BLI. Kaplan-Meier plots of survival. TTI-621 significantly inhibited tumor growth by both BLI (*p* = 0.001) and animal survival (*p* = 0.0003). *≤0.05, **≤0.01, ***≤0.001, ****≤0.0001. Two-Tailed *t*-Test Assuming Equal Variances. Error bars represent the mean +/− standard error of the mean.

Given that myeloid cells are prominent in tumor microenvironment and that *CD47* expression correlated with worse outcome, lower response to therapy and an immunosuppressive tumor microenvironment, it was of interest to assess the anti-tumor efficacy of anti-CD47 therapy alone and in combination especially with PARP inhibition or antibody drug conjugates. Therefore, we then investigated the activity of anti-CD47 therapy both in-vitro and in-vivo in ovarian cancer models. TTI-621 is a human SIRPa-Fc (hIgG1) decoy receptor that interrupts CD47-SIRPa interactions. Compared to control, CD47 blockade with SIRPa-Fc decoy receptor (TTI-621) enhanced macrophage-mediated in-vitro phagocytosis of ovarian cancer cells (OVCAR3 and TOV-21G) (Fig. 2A and Supplementary Fig. 4). We then investigated the activity of anti-CD47 in in-vivo xenograft models of ovarian cancer. The anti-tumor activity of the anti-CD47 blocking antibody, Hu-5F9, was assessed (compared to control and to Olaparib) in a PDX model of chemotherapy resistant, PARP inhibitor resistant high grade serous ovarian cancer. The PDX tumor was implanted subcutaneously in NSG mice and treatment was started once tumor was established. Anti-CD47 therapy showed significant anti-tumor activity compared to control and was superior to Olaparib (*p* < 0.001) (Fig. 2B). Then we assessed the activity of CD47 blockade therapy with SIRPa-Fc decoy receptor (TTI-621) using intraperitoneal (IP) xenograft ovarian cancer models with three ovarian cancer cell lines (TOV-21G, OVCAR3 and SKOV3). NOD/SCID mice were intraperitoneally implanted with luciferase expressing ovarian cancer cells and treatment was administered as outlined in Fig. 2C, D and E. Anti-tumor activity was assessed by measuring bioluminescence. TTI-621 significantly inhibited tumor growth in TOV-21G (Fig. 2C), OVCAR3 (Fig. 2D) and SKOV3 (Fig. 2E) with improved survival compared to control. No toxicity was observed for mice treated with TTI-621 as measured by body weight change (data not shown).

### Anti-CD47 synergized with PARP inhibition in BRCA deficient ovarian cancer xenograft models and enhanced macrophage-mediated in-vitro phagocytosis

**Fig. 3:**
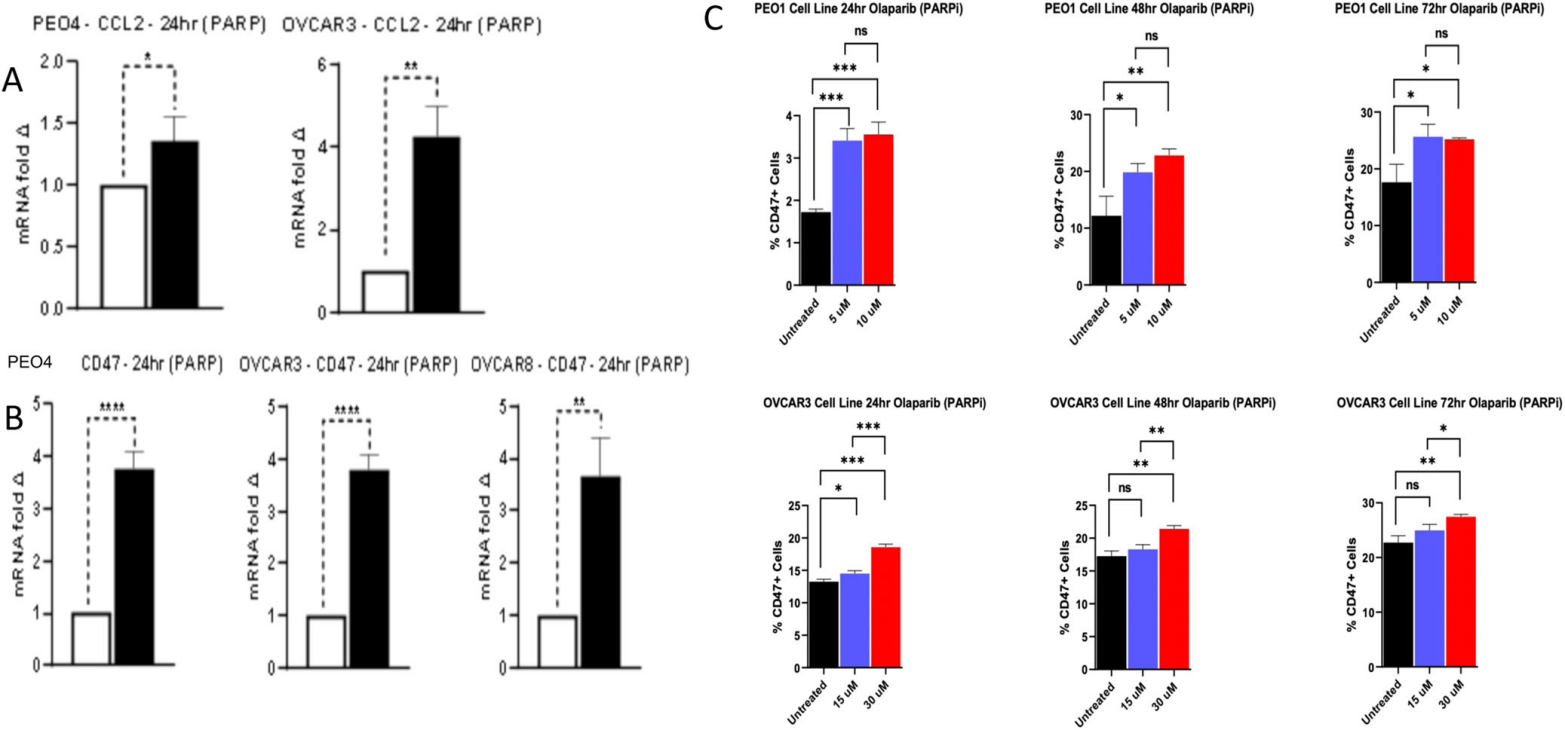
Expression analyses of different ovarian cancer cells. Gene expression analysis of CD47 and CCL2 was with RT-QPCR of ovarian cancer cells and CD47 protein expression analysis with flow cytometry were performed. For gene expression analyses, ovarian cancer cells were treated in-vitro with olaparib for 24 h using IC50. For flow cytometry, ovarian cancer cells were treated for 24, 48 and 72 h. **A** *CCL2* expression in ovarian cancer cells treated with olaparib vs. untreated. **B** *CD47* expression in ovarian cancer cells treated with olaparib vs. untreated. **C** CD47 expression on surface of cancer cells was assessed by flow cytometry after 24 h, 48 h and 72 h of treatment with olaparib compared to control. For analyses, two tailed *p*-value was calculated assuming equal variance (*≤0.05, **≤0.01, ***≤0.001, ****≤0.0001). Error bars represent the mean +/− standard error of the mean.

**Fig. 4:**
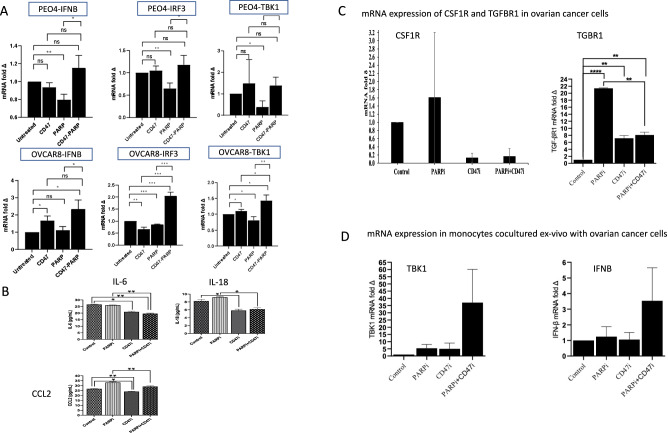
Expression analyses of different ovarian cancer cells. Gene expression analysis with RT-QPCR of ovarian cancer cells and cytokine analyses using meso-scale assay. **A** Ovarian cancer cells were treated in-vitro with olaparib, anti-CD47 (Hu-5F9) or both for 24 h using IC50 and RNA was extracted after that. Gene expression of markers of STING pathway (*TBK1, IRF3* and *IFNB*) in PEO4, *BRCA2* mutated chemotherapy resistant ovarian cancer cells and in OVCAR8, *BRCA2* wild type ovarian cancer cells. **B** Cytokines levels in supernatant of in-vitro experiment using PEO4 cancer cells after 96 h treatment with olaparib, anti-CD47 (Hu-5F9) or both compared to control. **C** Ovarian cancer cells were treated in-vitro with olaparib, anti-CD47 (Hu-5F9) or both for 24 h using IC50 and RNA was extracted after that. Gene expression of markers of immunoupressoe myeloid phenotype were assessed including CSF1R and TGFB1R in PEO4 ovarian cancer cells. **D** Co-culture experiment of UWB1.289.BRCA1 ovarian cancer cells (BRCA1 mutated with reversion mutation) with monocytes using indirect trans-well culture system with 48 h treatment followed by RNA extraction from monocytes. Gene expression of markers of STING pathway (*TBK1*and *IFNB*) were assessed. For analyses, two tailed *p*-value was calculated assuming equal variance (*≤0.05, **≤0.01, ***≤0.001, ****≤0.0001). Error bars represent the mean +/− standard error of the mean.

We then assessed the impact of PARP inhibition using Olaparib on *CD47* expression and *CCL2* expression (known to be up-regulated by chemotherapy and recruit tumor associated myeloid cells). We noted that Olaparib significantly up-regulated *CD47* and *CCL2* expression in ovarian cancer cells in-vitro (Fig. 3A and B and Supplementary Fig. 5). Then we sought to assess the impact of CD47 inhibition on STING pathway by assessing gene expression of *TBK1* and other downstream markers in STING pathway including *IRF3* and IFN-β. Ovarian cancer cells were treated with anti-CD47 (Hu-5F9), Olaparib or both. Anti-CD47 therapy showed evidence of up-regulation of markers of STING pathway especially when combined with Olaparib in ovarian cancer cells (Fig. 4A). Combined anti-CD47 therapy with olaparib also reduced cytokine secretion of IL6, IL18 and CCL2 from ovarian cancer cells (Fig. 4B). We also noted that anti-CD47 when combined with olaparib downregulated immunosuppressive myeloid markers in cancer cells specifically, TGFBR1 and CSF1R, which has been shown to correlate with therapy resistance (Fig. 4C). Then we showed that anti-CD47 therapy combined with olaparib also upregulated STING pathway markers (TBK1 and IFNB) in monocytes cocultured with ovarian cancer cells (Fig. 4D).

Given prior studies that reported synergy with cytotoxic agents, we then investigated the activity of anti-CD47 therapy combined with PARP inhibition compared to control and monotherapy both in-vitro and in-vivo in *BRCA* mutated ovarian cancer models. Compared to control, CD47 blockade therapy with SIRPa-Fc decoy receptor (TTI-621) enhanced macrophage-mediated in-vitro phagocytosis of ovarian cancer cells (*BRCA1* mutated, UWB1.289 and its counter cell line with *BRCA1* wild type, UWB1.289). The activity of anti-CD47 combined with PARP inhibition was greater in the BRCA1 deficient cells compared to BRCA wild type cells for both veliparib and niraparib (Fig. 4A).

Then we sought to investigate whether combined anti-CD47 therapy and PARP inhibition will be more effective than monotherapy using two in-vivo xenograft models: (1) *BRCA2* mutated PEO1 ovarian cancer cells implanted subcutaneously in NSG mice and (2) SKOV3 with BRCA1 knockdown (KD) implanted intraperitoneally in NOD/SCID mice. Combined anti-CD47 synergized with PARP inhibition in both models compared to control and PARP inhibitor monotherapy. In the first model, anti-CD47, Hu-5F9 was added to Olaparib in comparison to Olaparib monotherapy. Combined therapy significantly enhanced anti-tumor activity with complete cure of the mice (Fig. 4B). In the second model, SKOV3 (luciferase positive, BRCA1 KD, Fig. 4C) cells were implanted intraperitoneally in the NOD/SCID mice and treated with combined TTI-621 and niraparib vs. monotherapy and control. Combined therapy significantly enhanced the anti-tumor activity of niraparib and prolonged the survival of the mice (Fig. 4D). TTI-621 treatment was well tolerated with no negative impact on body weight.

We also sought to investigate whether combined anti-CD47 therapy and the anti-HER2 ADC, T-DM1 would be more effective than monotherapy using two in-vivo xenograft models: (1) A *BRCA1* mutated high grade serous ovarian cancer model (chemotherapy and PARP inhibitors resistant) that exhibits HER2 and MYC amplification, (2) A very aggressive chemotherapy naïve uterine serous carcinoma model that exhibits *HER2* and *CCNE1* amplification as well as *TP53* and *PIK3CA* mutations. In both models, cancer cells/tumors were implanted subcutaneously in NSG mice and treatment started when tumor was established. In the PDX model, combined anti-CD47 synergized with T-DM1 and significantly enhanced the anti-tumor activity of the combined regimen combined to monotherapy and control (Supplementary Fig. 6A). In the second model, anti-CD47 showed additive effect when combined with T-DM1 and enhanced the anti-tumor activity when combined anti-CD47 + T-DM1 compared to T-DM1 alone or paclitaxel (Supplementary Fig. 6B). Treatment was well tolerated with no negative impact on mice.

## Discussion

CD47 is a “do not eat me signal” that serves as an innate immune checkpoint, allowing tumor cells to evade macrophage-mediated phagocytosis. CD47 has been shown to be highly expressed in many solid tumors and predict survival in these tumors^7^. Using TCGA data in our prior study, we have shown that high *CD47* expression correlates with poor progression-free survival in ovarian and endometrial cancers^3^. In this study, we showed that high *CD47* gene expression correlated with lower infiltrating cytotoxic T-cells and dendritic cells as well as lower *PDL1* and *CTLA4* expression. These data suggest that CD47 might contribute to an immunosuppressive tumor immune microenvironment with lower activated T-cells and antigen presenting cells. Further, higher *CD47* expression could be a proxy of predominant immunosuppressive tumor associated macrophages. In fact, tumor associated myeloid cells and neutrophils were the most predominant cells in the tumor immune environment in this cohort.

In this study, we performed transcriptomic RNA sequencing analysis of the tumor immune microenvironment in patients with gynecologic cancers who received immunotherapy with immune checkpoint inhibitors and stratified them based on immunotherapy outcome. *CD47* expression was significantly elevated in patients who failed immunotherapy with immune checkpoint inhibitors in the entire cohort and similar pattern in ovarian cancer sub-cohort. Additionally, patients with high CD47 levels tended to have shorter progression-free survival while on immunotherapy. This suggests that CD47 may play a role in making gynecologic cancers, including ovarian cancer, more resistant to immunotherapy treatments. In addition to enhancing macrophage-mediated phagocytosis and activating innate immune responses, another proposed mechanism of action for anti-CD47 therapy is the promotion of a pro-inflammatory phenotype of macrophages (M1 over M2), thereby enhancing antigen presentation by macrophages and dendritic cells leading ultimately to cross-priming and activation of T-cells mediated adaptive immune response. Thus, investigating the combined approach of targeting CD47 and immune checkpoint inhibition has been proposed. Some preclinical studies and early-stage clinical trials have shown promising results, with the combination therapy leading to increased tumor regression and improved survival in certain cancer types^17–20^. In fact, the activation of T-cells by immune checkpoint inhibition increased the effect of anti-CD47 signaling and immune related activity. In syngeneic immunocompetent models of colon cancer (CT26 and MC-38), anti-CD47 was synergistic with anti-PD1 or anti-PDL1 therapy. The combined therapy was more effective than monotherapy with significant inhibition of tumor growth. Further, the combined therapy was associated with increased activity of effector memory, central memory and cytotoxic CD8 and CD4 cells^17^. The combined regimen was associated with a switch in the macrophage phenotype with a 2.5 fold increase in the ratio of M1 to M2 macrophages and also decreases in monocytic myeloid derived suppressive cells (mMDSC)^17^, where anti-PD1 therapy alone did not affect tumor-associated macrophages. However, more research is needed to determine the optimal treatment regimens, potential side effects, and efficacy in various cancer types.

We demonstrated that *CD47* expression correlated positively with other factors that favor an immunosuppressive immune environment and therapy resistance such as TGF-β, BRD4 and CXCR4/CXCL12 pathways. TGF-β plays a significant role in suppression of antitumor immune responses and cancer progression. TGF-β has a significant impact on recruitment and promotion of M2 TAMs and has been shown to induce polarization toward M2 TAM phenotype via upregulation of the SNAIL pathway^21^. Further, CCL2 production has been shown to be regulated by TGF-β during breast cancer progression^22,23^. TGF-β has been shown to regulate the CCL2 gene promoter region^23^ and TGF-β inhibition significantly reduces CCL2 and CCL22 expression and subsequently cancer metastasis^22^. Therefore, high TGF-β within the TME can block development of M1 TAMs and induce formation and activation of an M2 immunosuppressive phenotype. In our prior study, we showed that TGF-β mediates immunotherapy resistance to ICI and predicts lower PFS especially in ovarian cancer^24^. In this study, we showed that both CD47 and TGF-β correlate with each other positively and mediate immunotherapy failure. Therefore, dual targeting of CD47 and TGF-β could have a synergistic effect on TAMs and need to be investigated preclinically.

Another interesting finding in our study is the positive correlation of *CD47* expression and *BRD4*. Prior studies have shown that *MYC* and *BRD4* genes regulate *CD47* expression at the transcription level^25^. High levels of MYC bind to the *CD47* gene promoter region and regulate its expression^25^. On the other hand, disruption of BRD4 binding to the super enhancer (SE) region of *CD47* has been shown to reduce *CD47* expression by disruption SE enhancing function^26^. In fact, inactivation of one of these genes or pharmacologic bromodomain and extra terminal proteins (BET) inhibition leads to decreased CD47 expression and enhanced anti-tumor immune responses^25,26^.

In this study, anti-CD47 therapy showed effective anti-tumor activity as a monotherapy in preclinical xenograft models of ovarian cancer as shown previously by Willingham et al. ^7^. We noted that PARP inhibition induces significant up-regulation of CD47 “do not eat me signal”. Further, anti-CD47 significantly enhanced macrophage-mediated phagocytosis of ovarian cancer cells in-vitro. Interestingly in the phase I doses escalation trial for Hu-5F9, 2 partial responses were noted in heavily pre-treated patients with recurrent ovarian cancer and one prolonged stable disease also occurred in a patient with ovarian cancer. These data are encouraging and support the potential role for targeting CD47 in ovarian cancer^27^. On the other hand, the phase Ib trial of magrolimab and anti-PDL1 in ovarian cancer showed no evidence of objective responses in 24 patients. Therefore, our focus was on other novel combinations with potential synergy. Overall early phase trials showed that these molecules targeting CD47-SIRPa axis are well tolerated with good safety profile. We are still awaiting more data in clinical efficacy and the proper combination approach in solid tumors including ovarian cancer.

We investigated the activity of anti-CD47 with PARP inhibitors (PARPi) both in-vitro and in-vivo and noted synergistic effect with the combination in *BRCA* mutated ovarian cancer models. PARP inhibitors are known to induce DNA damage and innate immune responses through activation of the STING pathway, especially in *BRCA* mutated tumors. Further, recent data have shown that tumor associated macrophages mediate resistance to PARPi in *BRCA* mutated triple negative breast cancer^20^. Our data support the potential role of targeting anti-CD47 in inducing STING pathway as a mechanism of synergy with PARP inhibition. This needs to be explored in future clinical trials.

The combination of anti-CD47 with an anti-HER2 antibody drug conjugate (ADC) such as T-DM1 represents an attractive approach for multiple reasons. First, as discussed above given the synergy with cytotoxic therapy, antibody drug conjugates such as T-DM1 have been shown to induce immunogenic cell death and increase DAMP signals including ATP, and calreticulin^28^. These molecules when expressed on the cancer cell surface provide “eat me” signals that enhance the phagocytic activity of macrophages and stimulate dendritic cells, leading to enhanced antigen presentation and cross-priming of T-cells. Calreticulin is a dominant pro-phagocytic signal by macrophages through interaction with its receptor (LRP1). This pro-phagocytic signal is counter-balanced by CD47. Consequently, cell surface calreticulin expression on tumor cells positively correlates with degree of anti-CD47 activity. Further, increased/additional calreticulin (CRT) is associated with increase anti-CD47 mediated phagocytosis^29^. These data support the combination regimen that we are proposing. Second, ADC molecules were shown to synergize with immune checkpoint inhibitors including anti-PD1 and anti-CTLA4 therapy^30,31^. Third, anti-CD47 has been shown to synergize with trastuzumab monoclonal antibody, augment tumor associated macrophages and phagocytosis as well as antibody-dependent cellular cytotoxicity (ADCC) activity^32^. Therefore, we investigated the pre-clinical activity of anti-CD47 therapy with anti-HER2 ADC, T-DM1 in two in-vivo xenograft models. In both models, anti-CD47 therapy significantly enhanced the anti-tumor activity of T-DM1.

Our study is limited by its retrospective nature and relatively small sample size. However, our study is one of the few that investigated the transcriptomic profile of the tumor immune microenvironment in gynecologic cancer patients treated with immunotherapy. Further, we utilized immune deficient mouse model which might have affected the magnitude of the benefit. The xenograft model does not reflect the complexity of human immune responses to cancer, therefore may not accurately represent the tumor microenvironment and interactions between the tumor and surrounding tissues. Moreover, differences in drug metabolism and pharmacokinetics between mice and humans can lead to inconsistent results in drug efficacy and toxicity studies. While our findings are encouraging, further investigation is necessary to confirm our results in larger preclinical studies and in human clinical trials.

In summary, our study sheds light on the potential of anti-CD47 therapy as a potential approach to overcoming immunotherapy resistance and improving responses to PARP inhibitors in ovarian cancer. If successful, this approach could lead to the development of novel combination therapies that improve outcomes for patients with ovarian cancer.

## Methods

### Patients population and tumor samples

Our patient cohort included endometrial, cervical, and ovarian cancers. Patient characteristics and available clinical and treatment data of our patients stratified by responses to immune checkpoint inhibition (ICI) are summarized in Supplementary Table 1. The study received ethical approval by Institutional review Board at Cleveland Clinic and University of Pittsburgh.

Patients were classified as responders if they had evidence of a decrease in radiologic tumor burden with partial or complete response by RECIST 1.1 when evaluated by CT scan per judgment of their treating physician. Patients had to have at least 2 cycles of immunotherapy with interval imaging to assess response compared to pre-treatment imaging. The details and frequency of immune related toxicities were collected retrospectively from patient’s medical records. The progression-free survival was calculated from time of initiation of immunotherapy with immune checkpoint inhibition to disease progression, last follow up or death. This was done to elucidate the impact of immunotherapy on the progression-free survival endpoint.

Formalin-fixed paraffin-embedded (FFPE) tumor samples were collected from the 47 patients that had been enrolled into this retrospective study with Cleveland Clinic institutional review board approval. The archival FFPE samples were collected when sufficient (>20% tumor content) tumor material was available. Waiver of informed consent was obtained because de-identified residual tissue was used from pathology department.

### Next-generation sequencing and bioinformatics pipeline

FFPE specimens were processed and sequenced by MedGenome (Foster City, CA). The archival FFPE samples were used for sequencing when sufficient (>20% tumor content) tumor material was available. All 49 specimens passed RNA extraction QC and RNA library prep QC and proceeded for mRNA sequencing. Based on quality report of fastq files we trimmed sequence reads wherever necessary to only retain high quality sequence for further analysis. In addition, the low-quality sequence reads were excluded from the analysis. Data quality checks were performed using FastQC (v0.11.8). The paired-end reads were aligned to the reference Human genome Feb. 2009 release downloaded from UCSC database (GRCh37/hg19). The chromosome fasta file was downloaded from the following website (http://hgdownload.soe.ucsc.edu/goldenPath/hg19/bigZips/chromFa.tar.gz). GTF file was downloaded from the following website (ftp://ftp.ensembl.org/pub/release75/gtf/homo_sapiens/Homo_sapiens.GRCh37.75.gtf.gz). Alignment was performed using STAR (v2.7.3a)^33^. The aligned reads were used for estimating expression of the genes using HTSeq (v0.11.2). Only reads mapping unambiguously to a single gene were counted, whereas reads aligned to multiple positions or overlapping with more than one gene were discarded. Read count data were normalized and gene expression analysis was performed using R/Bioconductor packages DESeq2 (v1.28.1)^34^. Pathway analysis, gene signature, and upstream regulators were identified using QIAGEN Ingenuity Pathway Analysis (IPA, QIAGEN, Redwood City, CA).

### CD47 expression score validation

We evaluated CD47 expression score for its predictive power of response outcome and overall survival using online tool TIDE: Tumor Immune Dysfunction and Exclusion, which applies custom biomarker gene set to gene expression profiles of 23 cancer studies with immunotherapy, and compared results to existing published biomarkers^35^(http://tide.dfci.harvard.edu).

### Gene signature score and immune cell abundance estimation

The signature-based scoring was calculated using the rank-based single-sample gene set scoring method (simpleScore) provided by R package^36^. Singscore method implements a simple single-sample gene-set (gene-signature) scoring method which scores individual samples independently without relying on other samples in gene expression datasets.

### Immune cell abundance estimation

Population abundance of tissue-infiltrating immune and stromal cells were estimated using MCPcounter R package^11^ from bulk transcriptome data. The program predicts the abundance of 10 cell populations (8 immune populations together with endothelial cells and fibroblasts) from transcriptomic profiles of human tissues. MCP-counter estimates are “single sample” scores, which calculate score for each single sample. MCP-counter scores were defined as the log2 average expression of the transcription marker gene for each population. Genes used for cell abundance estimation were listed in Supplementary Table 1.

### Integrative model development

We implemented a random forest classifier using the random Forest package (version 4.7-1.1) in R programming language (ref. ^37^). The final model has number of trees =500, number if variables tried at each split =5. With 41 samples having complete response information included in the model, the out of bag (OOB) error rate was estimated as 19%. The variable importance was estimated by randomForest and plotted with varImpPlot function as a dotchart for Gini coefficient, which is a measure of how each variable contributes to the homogeneity of the nodes and leaves in the final random forest model. The higher the value of mean decrease Gini score, the higher the importance of the variable in the model.

### Reagents

Anti-CD47 molecules including Hu-5F9 and TTI-621 were obtained from Forty Seven, Inc. and Trillium Therapeutics Inc., respectively. Olaparib was purchased from Selleckchem. T-DM1 were obtained from institutional clinical Pharmacy. The antibodies were purchased from Cell Signaling.

### Cell lines and cell cultures

ARK1 cell line are uterine serous carcinoma cell line that is characterized by TP53, PIK3CA mutations, HER2 and CCNE1 amplifications. It is established from a stage IV chemonaive patient. This cell line was obtained from Dr. Santin at Yale School of Medicine and was grown in RPMI with 10% FBS and 0.3% anti-fungal. PEO1 is a *BRCA2* mutated ovarian cancer cell line and was purchased from Sigma-Aldrich. UWB1.289.BRCA1, OVCAR3 and TOV-21G are ovarian cell lines and were purchased from ATCC.

**Fig. 5:**
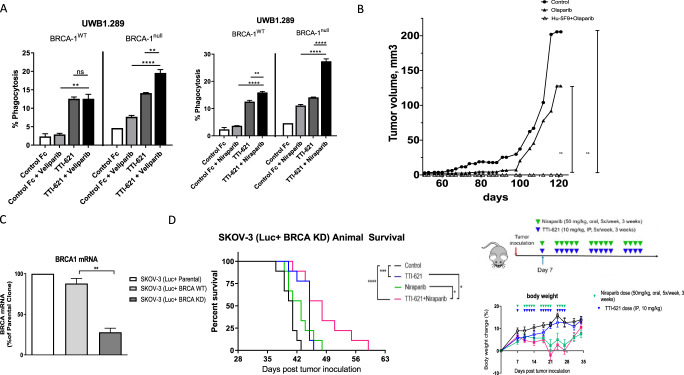
Preclinical in-vitro and in-vivo activity of anti-CD47 therapy with PARP inhibition in ovarian cancer cells. **A** in-vitro phagocytosis of Veliparib, Niraparib or untreated UWB1.289 BRCA-1WT and BRAC-1Null cells with macrophages in the presence of 1 uM TTI-621 or control Fc. **B** In-vivo anti-tumor activity of anti-CD47 therapy with Hu-5F9 combined with Olaparib compared to control and Olaparib in CDX model of high grade serous ovarian cancer with *BRCA2* mutation (PEO2 ovarian cancer cells). Ovarian cancer cells were implant subcutaneously; treatment was started when tumor was established. Anti-CD47 was given intraperitoneally twice a week for four weeks. Olaparib was given 50 mg/kg oral gavage 5 times a week till end of the experiment. **C** *BRCA1* RNA expression in SKOV3 *BRCA1* wild type and *BRCA1* KD cell lines. RNA was extracted from cell lines, followed by cDNA synthesis by reverse transcription. Expressions of *BRCA1* were quantified using qPCR, and relative expressions of *BRCA1* WT and *BRCA1*-KD cells to the parental SKOV3 Luc+ cells are shown. **D** NOD/SCID mice implanted (IP) with SKOV-3 (Luc+BRCA1 KD) and treated with TTI-621, niraparib, or TTI-621+niraparib. TTI-621 significantly inhibited tumor growth and prolonged survival (***p < 0.001) compared to vehicle control. TTI-621 + niraparib is superior to either monotherapy (*p* = 0.0436 compared to TTI-621 alone, and *p* = 0.0105 compared to niraparib alone). Body weight change of animals with no difference between groups. *≤0.05, **≤0.01, ***≤0.001, ****≤0.0001. Two-Tailed *t*-Test Assuming Equal Variances. Error bars represent the mean +/− standard error of the mean.

SKOV3 is an ovarian cancer cell line that is characterized by *TP53* mutation, *HER2* amplification and *PIK3CA* mutation. SKOV3 was purchased from Cell Biolabs Inc. SKOV3 cells were cultured in DMEM with low glucose, supplemented with 10% Fetal Bovine Serum, penicillin and streptomycin. All cell lines were confirmed negative for mycoplasma and maintained at 37 °C in a humidified atmosphere at 5% CO2. SKOV3-Luciferase expressing cells (SKOV3 Luc+) were generated by lentivirus (CMV-firefly luciferase-GFP) transduction followed by cell sorting. SKOV3-BRCA knockdown cell line was established. Using SKOV3 Luc+ cell line as described, BRCA1 was knocked down using lentiviral shRNA constructs (gene target sequence GAGAGTGCTTGGGA-TCGAT). Control shRNA was used to generate a BRCA1 competent cell line (Luc+ BRCA+). BRCA1 knock-down cells (Luc+ BRCA KD) have significantly lower BRCA1 expression compared to SKOV3 (Luc+ BRCA1+) (*p* = 0.0016) (Fig. 5C). The expression level of BRCA1 was performed by qPCR using cDNA generated from SKOV3-BRCA1 KD and control cells. Data is represented as percentage of expression relative to SKOV3 Luc+ parental clone.

Quantitative Real-Time PCR (qRT-PCR) was carried out using cybergreen probes from Applied Biosystems (BioRad), according to the manufacturer’s recommendations. Reactions were carried out in an ABI 7000 sequence detector (Perkin Elmer) and results were expressed as fold change calculated by the ΔC_t_ method relative to the control sample. GAPDH was used as an internal normalization control. Measurement was done in duplicates. See Supplementary Table 3 for sequences of the primers.

A patient-derived xenograft (PDX) model of high-grade serous ovarian cancer, platinum resistant that was established by Dr. Mahdi. This PDX model is characterized by *BRCA1* and *PIK3CA* mutation and *HER2* amplification. This PDX is established from patient with platinum-resistant and PARP inhibitors resistant disease at time of tertiary debulking surgery.

PBMC from normal donors were purchased from BioIVT and written informed consent was obtained from all donors compiling with all institutional ethical regulation. CD14+ monocytes were isolated from PBMCs by positive selection using human monocyte isolation kit. Monocytes were differentiated into macrophages by culturing for at least ten days in X-Vivo-15 media (Lonza) supplemented with M-CSF (PeproTech). One day before the phagocytosis assay, macrophages were primed with IFNg (PeproTech). On the day of the phagocytosis assay, macrophages were co-cultured with violet proliferation dye 450 (VPD450)-human ovarian cell line OVCAR-3 or TOV-21G or UWB1.289 in the presence of TTI-621 for two hours. Phagocytosis was assessed as % VPD450+ cells of live, single CD14 + CD11b+ macrophages by flow cytometry. In the in vitro PARP inhibitor combination experiment, UWB1.289 cell line was treated with 10uM Veliparib (Selleckchem) or 10uM Niraparib (MedChemExpress) for three days before the phagocytosis assay.

Ex-vivo coculture experiments was performed where monocytes were cocultured with ovarian cancer cells using indirect trans-well system. Cells were plated overnight and next day, treatment was applied for 48 h then after that cells and supernatant were collected for analyses.

### Cytokine analysis

Quantification of cytokines present in the supernatant from ovarian cancer cells treated in-vitro with olaparib, Hu-5F9 or both was performed using the Meso Scale Discovery Multi-Spot Assay System (Meso Scale Diagnostics, LLC). V-PLEX assays were performed per manufacturer’s instructions. Briefly, MULTI-SPOT assay plates pre-coated with capture antibodies were washed and incubated with diluted supernatant fluid samples and/or calibrators for two hours at room temperature. Next, plates were washed and bound analytes measured using detection antibodies conjugated with electrochemiluminescent labels (MSD SULFO-TAG). Following an additional two hours incubation at room temperature, plates were washed, incubated with electrochemiluminescence buffer, and analyzed on a MESO QuickPlex SQ 120 instrument. Data was processed using MSD Discovery Workbench Version 4.0.

### Cell lines (CDX) and patient-derived xenografts (PDX) studies

NSG (NOD-SCID IL2Rγ−/−, Bar Harbor, ME) and NOD/SCID mice were purchased from the Jackson lab. This study received ethical approval and was carried out according to the policies of the Institutional Animal Care and Use Committee (IACUC) at University of Pittsburgh. The study has complied with all relevant ethical regulations for animal testing and research.

Five-eight weeks old female mice were used for tumor transplantation. Ovarian cell lines were implanted subcutaneously (s.c.) (2 million cells per mouse) or intraperitoneally (i.p.) (5 million cells per mouse). For tumors implanted s.c., treatments were started 1 week after tumor implanted and established when the size of the tumor reaches in ~6 mm in diameter or 100–120 mm^3^ volume. For tumors implanted intraperitoneally, NOD/SCID mice were implanted with luciferase expressing cell lines including OVCAR3, TOV-21G, SKOV3 and SKOV3 BRCA1 KD. Tumor growth was monitored once every 1–2 weeks by Bioluminescence (BLI) at randomization and after treatment. Briefly, mice were injected intraperitoneally with 150 mg/kg of luciferin (Promega) at various times prior to BLI acquisition (IVIS Imaging System, Perkin Elmer, Waltham, Massachusetts) under isoflurane anesthesia. The results of BLI were reported as total photon flux/second (p/s).

Mouse weight and tumor size was recorded two times a week for the entire experimental period. Tumor volume will be calculated as a mean. Negative (vehicle) controls were utilized. Doses of the targeted therapy drugs were as follow: T-DM1 2 mg/kg ip 2x/week for 4 weeks, Olaparib 50 mg/kg oral gavage 5 times/week, Niraparib 50 mg/kg oral gavage 5 times/weeks for 3 weeks, Hu-5F9 200 mcg IP 2 times/week for 4 weeks, and TTI-621 10 mg/kg IP 5 times/weeks for 3 weeks. Patient-derived xenografts are generated by sectioning of fresh tumor tissue and engrafting pieces (2 × 2 × 2 mm^3^) subcutaneously. Tumor was obtained from tertiary debulking surgery conducted at the Cleveland Clinic (IRB#2017-1863). Written informed consent was obtained from the patient for the use of their tissues. Once the transplanted tissue reaches ~700–1000 mm^3^, it is harvested, analyzed by genomic and proteomic studies, expanded and banked for future studies. Tumor length and width were measured on each mouse and used to calculate tumor volume. Once tumor volume reached 70–100 mm^3^, animals (*n* = 56) were randomized to the treatment groups. Tumor volume and body weight were measured weekly. Animals were euthanized according to Institutional Animal Care and Use Committee guidelines. Tumors were collected and snap frozen for future analyses.

### Statistical analysis

We used the two-sided Student’s *t*-test test for all comparisons of continuous data and the Spearman correlation coefficient to analyze correlation between different variables. Fisher’s exact test was used to compare the signature score level between different groups. Kaplan–Meier estimation and log rank tests were used for time-to-event analyses comparing between 2 groups based on individual variables such as response and gene/score with the cohort median value used as cut-off. Survival analysis on continuous variables such as gene expression was performed using a multi-variable. The statistical significance for both pathway analysis and upstream regulator analysis was assessed via Fisher’s exact test by IPA.

All statistical tests were two-sided, and a *p* value of less than 0.05 was considered significant across all analyses performed. Statistical analyses were performed using R (version 4.0.3) and RStudio (version 1.3.1093). For gene expression analyses, two tailed *p*-value was calculated assuming equal variance (*≤0.05, **≤0.01, ***≤0.001, ****≤0.0001).

An experimental group size of 8 tumors per group is expected at a power of 80% to detect a tumor volume difference between animals with *p* < 0.05. For each independent experimental group, the measures of growth were normalized to the mean of the control group, so that all data are expressed as a proportion of the control. ANOVA test was to determine the statistical significance of the effects of combination treatment when compared to the control and to the single agent treatment. Differences in all comparisons were considered significant at *P* values < 0.05.

### Reporting summary

Further information on research design is available in the Nature Research Reporting Summary linked to this article.

## Supplementary information

Supplementary materials

REPORTING SUMMARY

## Data Availability

The RNA sequencing data used in the current study are deposited in SRA repository (SRP341153) with the following accession number: PRJNA770873, which will be available immediately following publication.

## Code availability

The codes and software packages are described in the methods section. Any further codes or other data are available upon request.

## References

[1] Gaillard SL, Secord AA, Monk B (2016). The role of immune checkpoint inhibition in the treatment of ovarian cancer. Gynecol. Oncol. Res Pr..

[2] Hamanishi J (2015). Safety and antitumor activity of anti-PD-1 antibody, nivolumab, in patients with platinum-resistant ovarian cancer. J. Clin. Oncol..

[3] Ni Y (2021). Immune cells and signatures characterize tumor microenvironment and predict outcome in ovarian and endometrial cancers. Immunotherapy.

[4] Gyori D (2018). Compensation between CSF1R+ macrophages and Foxp3+ Treg cells drives resistance to tumor immunotherapy. JCI Insight.

[5] Zhang M (2014). A high M1/M2 ratio of tumor-associated macrophages is associated with extended survival in ovarian cancer patients. J. Ovarian Res..

[6] Lyons YA (2017). Macrophage depletion through colony stimulating factor 1 receptor pathway blockade overcomes adaptive resistance to anti-VEGF therapy. Oncotarget.

[7] Willingham SB (2012). The CD47-signal regulatory protein alpha (SIRPa) interaction is a therapeutic target for human solid tumors. Proc. Natl Acad. Sci. USA.

[8] Tseng D (2013). Anti-CD47 antibody-mediated phagocytosis of cancer by macrophages primes an effective antitumor T-cell response. Proc. Natl Acad. Sci. USA.

[9] Tsai RK, Discher DE (2008). Inhibition of "self" engulfment through deactivation of myosin-II at the phagocytic synapse between human cells. J. Cell Biol..

[10] Cioffi M (2015). Inhibition of CD47 effectively targets pancreatic cancer stem cells via dual mechanisms. Clin. Cancer Res..

[11] Becht E (2016). Estimating the population abundance of tissue-infiltrating immune and stromal cell populations using gene expression. Genome Biol..

[12] Mahdi H, Ni Y (2020). Immunogenomic Signatures to predict outcome in ovarian and endometrial cancers: potential strategies in tergeting the tumor immune microenvironment to improve response to immunotherapy. J. Clin. Oncol..

[13] Mahdi H (2019). The role of different TGFβ signatures in predicting outcome in high grade serous ovarian carcinoma. J. Clin. Oncol..

[14] Samanta D (2018). Chemotherapy induces enrichment of CD47(+)/CD73(+)/PDL1(+) immune evasive triple-negative breast cancer cells. Proc. Natl Acad. Sci. USA.

[15] Feliz-Mosquea YR (2018). Combination of anthracyclines and anti-CD47 therapy inhibit invasive breast cancer growth while preventing cardiac toxicity by regulation of autophagy. Breast Cancer Res Treat..

[16] Lo J (2016). Anti-CD47 antibody suppresses tumour growth and augments the effect of chemotherapy treatment in hepatocellular carcinoma. Liver Int..

[17] Kauder SE (2018). ALX148 blocks CD47 and enhances innate and adaptive antitumor immunity with a favorable safety profile. PLoS One.

[18] Lian S (2019). Simultaneous blocking of CD47 and PD-L1 increases innate and adaptive cancer immune responses and cytokine release. EBioMedicine.

[19] Lian S (2019). Dual blockage of both PD-L1 and CD47 enhances immunotherapy against circulating tumor cells. Sci. Rep..

[20] Tao H (2017). Targeting CD47 enhances the efficacy of anti-PD-1 and CTLA-4 in an esophageal squamous cell cancer preclinical model. Oncol. Res..

[21] Zhang F (2016). TGF-beta induces M2-like macrophage polarization via SNAIL-mediated suppression of a pro-inflammatory phenotype. Oncotarget.

[22] Mandal PK (2018). CCL2 conditionally determines CCL22-dependent Th2-accumulation during TGF-beta-induced breast cancer progression. Immunobiology.

[23] Gorbacheva AM (2021). EGR1 and RXRA transcription factors link TGF-beta pathway and CCL2 expression in triple negative breast cancer cells. Sci. Rep..

[24] Ni Y (2021). High TGF-beta signature predicts immunotherapy resistance in gynecologic cancer patients treated with immune checkpoint inhibition. NPJ Precis Oncol..

[25] Casey SC (2016). MYC regulates the antitumor immune response through CD47 and PD-L1. Science.

[26] Betancur PA (2017). A CD47-associated super-enhancer links pro-inflammatory signalling to CD47 upregulation in breast cancer. Nat. Commun..

[27] Sikic BI (2019). First-in-human, first-in-class phase I trial of the anti-CD47 antibody Hu5F9-G4 in patients with advanced cancers. J. Clin. Oncol..

[28] Bauzon M (2019). Maytansine-bearing antibody-drug conjugates induce in vitro hallmarks of immunogenic cell death selectively in antigen-positive target cells. Oncoimmunology.

[29] Chao MP (2010). Calreticulin is the dominant pro-phagocytic signal on multiple human cancers and is counterbalanced by CD47. Sci. Transl. Med..

[30] Muller P (2014). Microtubule-depolymerizing agents used in antibody-drug conjugates induce antitumor immunity by stimulation of dendritic cells. Cancer Immunol. Res..

[31] Martin K (2014). The microtubule-depolymerizing agent ansamitocin P3 programs dendritic cells toward enhanced anti-tumor immunity. Cancer Immunol. Immunother..

[32] Tsao LC (2019). CD47 blockade augmentation of trastuzumab antitumor efficacy dependent on antibody-dependent cellular phagocytosis. JCI Insight.

[33] Dobin A (2013). STAR: ultrafast universal RNA-seq aligner. Bioinformatics.

[34] Love MI, Huber W, Anders S (2014). Moderated estimation of fold change and dispersion for RNA-seq data with DESeq2. Genome Biol..

[35] Fu J (2020). Large-scale public data reuse to model immunotherapy response and resistance. Genome Med..

[36] Foroutan M (2018). Single sample scoring of molecular phenotypes. BMC Bioinforma..

[37] R Core Team. *R: A language and environment for statistical computing*. http://www.R-project.org/ (2013).

