# Supplementary material for: Targeting CD47-SIRPa axis shows potent preclinical anti-tumor activity as monotherapy and synergizes with PARP inhibition
Source: NPJ Precis Oncol. 2023 Jul 19;7:69. doi: 10.1038/s41698-023-00418-4 (PMC10356752; doi:10.1038/s41698-023-00418-4)

Supplementary Figure 1. A: Pair-wise correlation between *CD47*, another 10 immune markers, and TGF- β score (6-genes signature), with MCP immune cell abundance estimation in the entire cohort. Only statistical significant correlations were plotted out, non statistical significant correlations were displayed as blank grid. Positive correlation is in blue color and negative correlation in red color. B: Average expression levels of *CD47* , other immune markers included in our analysis, and MCP immune cell abundance estimation with objective response to immunotherapy with immune checkpoint inhibition (ICI).

Supplementary Figure 2. TIDE validation of *CD47* in multiple cancer datasets with immunotherapy. A: Our *CD47* as annotated "Custom" panel at the top, showed a good predictive value (AUC > 0.7) in 2 studies (Nathanson 2017_CTLA4_Melanoma_Pre and Kim2018_PD1_Gastric). B: Significant predictive value based on Coxph regression with *CD47* compared to other existing biomarkers in Gide2019_PD1+CTLA4_Melanoma study, with corresponding Kaplan Meier curve based on *CD47* median cut-off in the same study.

Supplementary Figure 3. Top differentially expressed genes and pathway compared patients with low and high *CD47* expression. *CD47* group "High" or "low" was defined by median RNA expression of *CD47*.

Supplementary Figure 4. Flow cytometric data showing gating strategy for the in-vitro phagocytosis experiments.

Supplementary Figure 5. Flow cytometric data showing %CD47 expression on tumor cells following treatment with olaparib at 48 hours compared to control in PEO1 and OVCAR3 cell lines.

Supplementary Figure 6. Preclinical in-vitro and in-vivo activity of anti-CD47 therapy with PARP inhibition in ovarian cancer cells. A: In-vivo anti-tumor activity of anti-CD47 therapy with Hu-5F9 combined with anti-HER2 ADC, T-DM1 compared to control or monotherapy in PDX model of HER2 amplified high grade serous ovarian cancer (chemotherapy and PARPi resistant model). PDX tumor was implanted subcutaneously, treatment was started when tumor was established. Anti-CD47 was given intraperitoneally twice a week for four weeks. T-DM1 was given 2 mg/kg IP twice a week for four weeks. B: In-vivo anti-tumor activity of anti-CD47 therapy with Hu-5F9 combined with anti-HER2 ADC, T-DM1 compared to control or monotherapy in CDX model of HER2 amplified high grade serous uterine cancer (ARK1 uterine serous cancer cells). Uterine serous cancer cells were implanted subcutaneously; treatment was started when tumor was established. Anti-CD47 was given intraperitoneally twice a week for four weeks. T-DM1 was given 2 mg/kg IP twice a week for four weeks. * ≤ 0.05, ** ≤ 0.01, *** ≤ 0.001, **** ≤ 0.0001. Two-Tailed t-Test Assuming Equal Variances

Supplementary table 1. Clinical characteristics of patients included in the study

Characteristic	No response	Response	Stable	p-value[2]
**DiseaseSite**				0.3
Ovarian	7 (33%)	4 (20%)	3 (50%)	
Endometrial	9 (43%)	14 (70%)	2 (33%)	
Cervical	5 (24%)	2 (10%)	1 (17%)	
**Histology**				0.003
adenocarcinoma	0 (0%)	0 (0%)	1 (17%)	
clear cell	0 (0%)	5 (25%)	0 (0%)	
endometrioid	5 (26%)	10 (50%)	1 (17%)	
mixed endometrioid and clear cell	0 (0%)	0 (0%)	1 (17%)	
serous	9 (47%)	3 (15%)	3 (50%)	
squamous	5 (26%)	2 (10%)	0 (0%)	
Unknown	2	0	0	
**Stage**				0.011
I	2 (10%)	10 (56%)	1 (20%)	
II	1 (5.0%)	1 (5.6%)	0 (0%)	
III	9 (45%)	3 (17%)	4 (80%)	
IV	8 (40%)	4 (22%)	0 (0%)	
Unknown	1	2	1	
**MSI**				0.2
Stable	7 (33%)	7 (35%)	1 (17%)	
High	5 (24%)	10 (50%)	2 (33%)	
Unknown	9 (43%)	3 (15%)	3 (50%)	
**Age**	69 (62, 75)	64 (62, 74)	68 (66, 72)	>0.9
Unknown	0	1	0	
**Cycles**	3 (3, 5)	12 (8, 16)	9 (8, 16)	<0.001
**PriorChemotherapyLines**				0.7
	0 (0%)	1 (5.0%)	0 (0%)	
0	2 (9.5%)	2 (10%)	0 (0%)	
1	6 (29%)	10 (50%)	3 (50%)	
11	1 (4.8%)	0 (0%)	0 (0%)	
2	3 (14%)	2 (10%)	1 (17%)	
3	5 (24%)	2 (10%)	0 (0%)	
4	1 (4.8%)	3 (15%)	1 (17%)	
5	1 (4.8%)	0 (0%)	0 (0%)	
7	1 (4.8%)	0 (0%)	1 (17%)	
8	1 (4.8%)	0 (0%)	0 (0%)	
**PlannedRegimen**				0.081
AVELUMAB	0 (0%)	1 (5.0%)	1 (17%)	
NIVOLUMAB	11 (52%)	4 (20%)	2 (33%)	
PEMBROLIZUMAB	10 (48%)	15 (75%)	3 (50%)	
**Toxicity**	4 (20%)	13 (65%)	4 (67%)	0.007
Unknown	1	0	0	
**Death**	18 (86%)	7 (37%)	2 (33%)	0.002
Unknown	0	1	0	
**OSMonth**	9 (4, 15)	17 (11, 23)	22 (17, 23)	0.008
Unknown	1	0	0	
**Progression**	19 (100%)	10 (53%)	2 (33%)	<0.001
Unknown	2	1	0	
**PFSMonth**	3 (2, 3)	11 (8, 17)	14 (9, 20)	<0.001
Unknown	2	0	0	

[1] n (%); Median (IQR)

[2] Fisher's exact test; Kruskal-Wallis rank sum test

Supplementary table 2. Multivariate analysis for variables associated with response to immunotherapy

Variable	Estimate	Std. Error	Z value	P value
CD47	-2.6674	1.2894	-2.069	0.0386
Cytotoxic lymphocytes	-0.1079	0.2401	-0.449	0.6533
Myeloid dendritic cells	-0.0558	0.2390	-0.233	0.8155

Supplementary table 3. Sequences of the primers

AGAAGGTGAAACGATCATCGAGC	CD47-F
CTCATCCATACCACCGGATCT	CD47-R
CAGCCAGATGCAATCAATGCC	CCL2-F
TGGAATCCTGAACCCACTTCT	CCL2-R
TTGGGCTGATCCTCTTCTTC	CSF1R-F
AAAGCGTGAGAGCACGAAGT	CSF1R-R
GAACTGTTTTGATTGGCATC	TGFBR1-F
AAGAAGGGACCTACACTATTT	TGFBR1-R
CGAAGCCGGAAGTGTCCTGAG	TBK1-F
CTCTGCATCTTGGCTGGATCA	TBK1-R
AACTTGCTTGGATTCCTACAAAG	IFNB-F
TATTCAAGCCTCCCATTCAATTG	IFNB-R
AGAGGCTCGTGATGGTCAAG	IFR3-F
AGGTCCACAGTATTCTCCAGG	IFR3-R
5′-ACAGCTGTGTGTGCTTCTGTG-3′	BRCA1-F
5′-CATTGTCCTCTGTCCAGGCATC-3′	BRCA1-R

Supplementary Figure 1

A

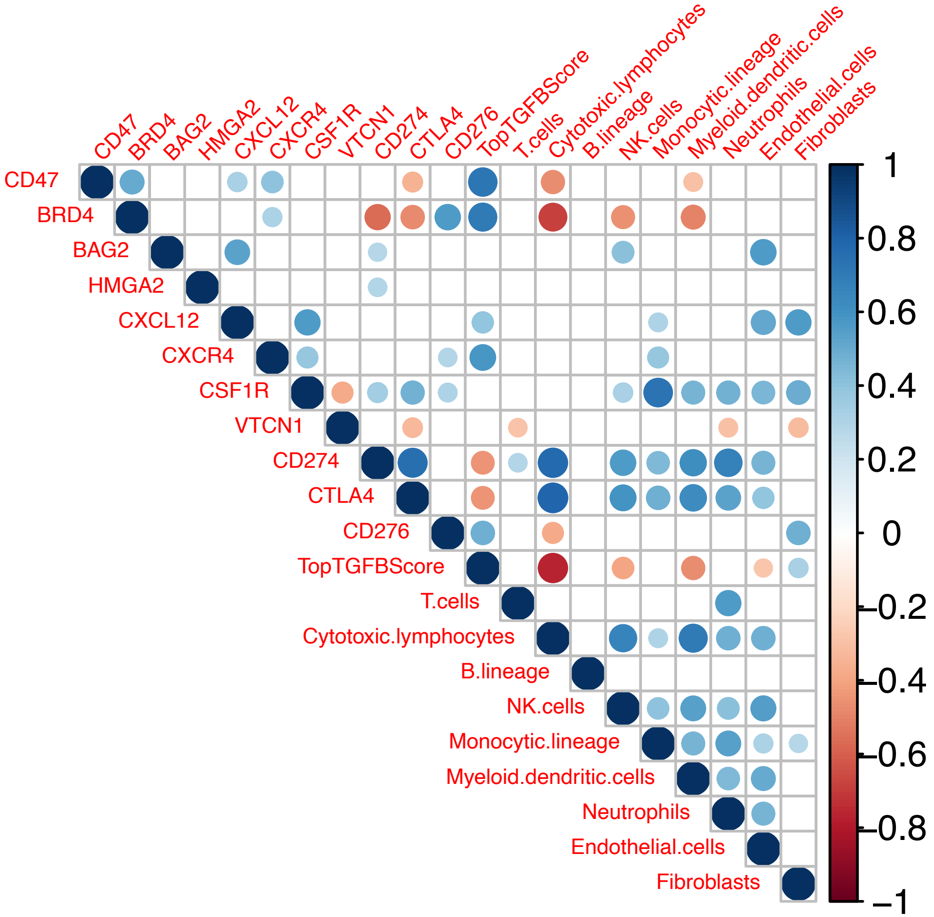

B

Based on Response

test.group	mean in group 0	mean in group 1	p.value
BRD4	0.904598167	0.828667259	0.33754427
BAG2	0.929742062	1.01761591	0.510191652
HMGA2	0.195472956	0.050916891	0.429250296
**CD47**	**1.360574656**	**1.142158002**	**0.033347339**
CXCL12	1.299211992	1.289765055	0.949662315
CXCR4	1.619388964	1.599492811	0.865471161
CSF1R	0.423522463	0.384983106	0.688613816
VTCN1	0.0980309	0.243838979	0.41985247
CD274	0.299762502	0.170947367	0.388039382
CTLA4	0.382602093	0.388283821	0.975308237
CD276	0.938868971	0.838689052	0.160415185
T.cells	8.039354987	4.034350236	0.20935408
Cytotoxic.lymphocytes	1.903950909	2.04416944	0.823415009
B.lineage	3.832530932	4.311712014	0.866192579
NK.cells	3.017378473	3.945511546	0.349763484
Monocytic.lineage	3.543971154	2.642301634	0.257516808
Myeloid.dendritic.cells	2.243743672	2.245971757	0.997105045
Neutrophils	4.033691138	3.751489273	0.611208341
Endothelial.cells	3.785660439	3.8267205	0.938096692
Fibroblasts	185.9755504	199.781001	0.825932328

Supplementary Figure 2

A

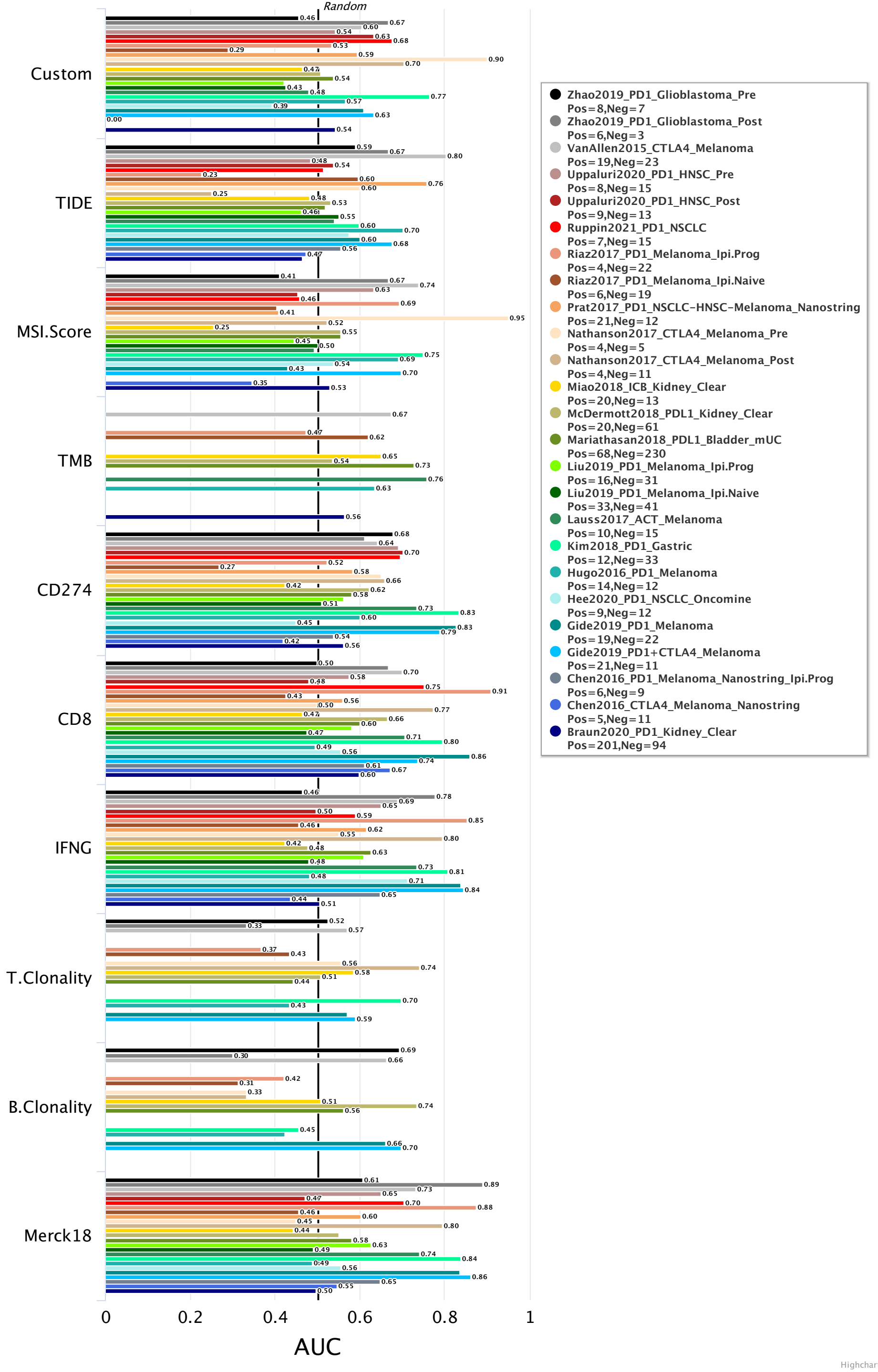

B

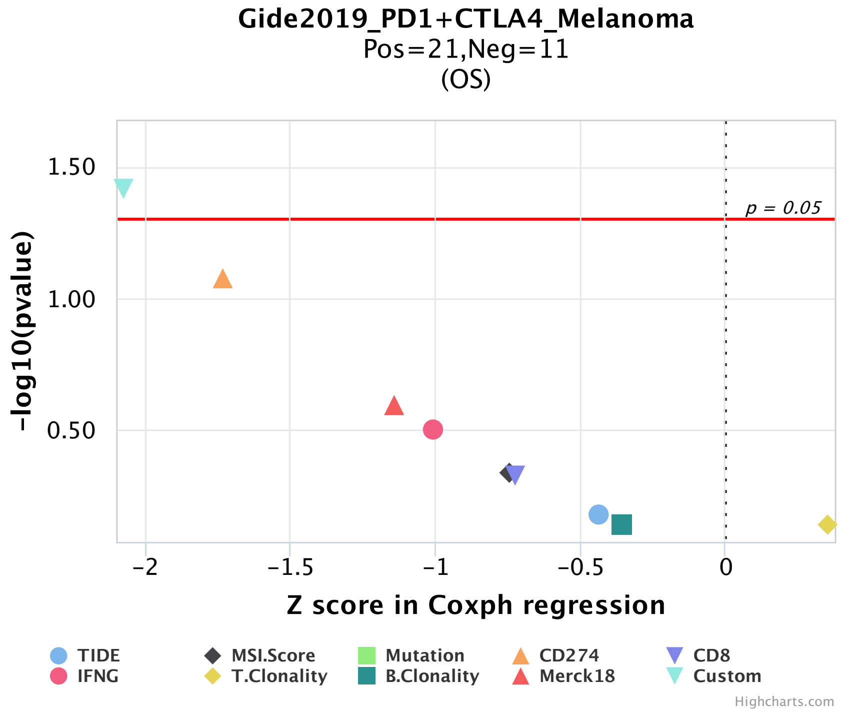

Custom: CD47

Supplementary Figure 3

DEG based on CD47 expression

CD47high vs Low: DE n=922 (padj < 0.05; FC>2)

log2FoldChai	lfcSE	stat	pvalue	padj	Symbol
3.08035551	0.40218368	7.65907628	1.87E-14	2.59E-10	ANK1
3.43088994	0.46860396	7.32151291	2.45E-13	5.64E-10	SHC4
1.00139281	0.14191645	7.05621372	1.71E-12	2.95E-09	DHRS7B
1.90618469	0.2725267	6.99448785	2.66E-12	3.77E-09	SEC16B
2.33348482	0.3344166	6.9777781	3.00E-12	3.77E-09	USHBP1
2.78643337	0.39905407	6.98259603	2.90E-12	3.77E-09	PXDNL
1.23211056	0.17739555	6.94555496	3.77E-12	4.22E-09	CCDC159
2.01596355	0.29449757	6.84543353	7.62E-12	6.58E-09	HEXIM2
2.06497962	0.30358819	6.8019103	1.03E-11	8.39E-09	LINC02983
2.19992498	0.32605233	6.74715314	1.51E-11	1.10E-08	LRRC66
3.04679976	0.45854772	6.64445507	3.04E-11	1.83E-08	ABCB4
2.438923	0.3674618	6.63721502	3.20E-11	1.84E-08	CACNB4
3.13815532	0.47348408	6.62779478	3.41E-11	1.88E-08	TMC1
1.72799074	0.26160812	6.60526418	3.97E-11	2.11E-08	LYG1
2.73645419	0.41781996	6.54936204	5.78E-11	2.96E-08	SCN2A
1.65121057	0.25302447	6.5258929	6.76E-11	3.30E-08	STOML1
1.52314204	0.23358319	6.520769	6.99E-11	3.30E-08	SLC25A45
2.60080687	0.40683734	6.39274373	1.63E-10	6.62E-08	PMEL
1.63204006	0.25632355	6.36710921	1.93E-10	7.60E-08	TMPRSS5
1.90519337	0.30104142	6.32867528	2.47E-10	9.23E-08	LOC646665
1.571913	0.24873373	6.31966155	2.62E-10	9.53E-08	DOHH
2.25694228	0.35786038	6.30676763	2.85E-10	1.01E-07	CDH26
1.41816209	0.22602638	6.27432116	3.51E-10	1.15E-07	STAC3
1.17888379	0.18809699	6.26742517	3.67E-10	1.17E-07	TNFSF13
2.07400401	0.3310542	6.26484729	3.73E-10	1.17E-07	RBMXL2
2.03319984	0.32495723	6.25682294	3.93E-10	1.19E-07	RINL
2.1290982	0.34070613	6.24907518	4.13E-10	1.19E-07	LOC400499
1.18857242	0.19079241	6.22966289	4.67E-10	1.27E-07	C1orf35
1.69892133	0.27318742	6.21888565	5.01E-10	1.30E-07	UCP3
1.3011604	0.20939613	6.21387026	5.17E-10	1.32E-07	CLN3
1.7356465	0.28176655	6.15987426	7.28E-10	1.62E-07	AGAP2
2.57778583	0.41948911	6.14506012	7.99E-10	1.75E-07	RPL34-DT
2.4393942	0.39784552	6.13151101	8.70E-10	1.85E-07	MSH4
2.6448584	0.43292587	6.1092639	1.00E-09	2.09E-07	PPP1R36
1.11356685	0.18251916	6.10109574	1.05E-09	2.17E-07	RGS3

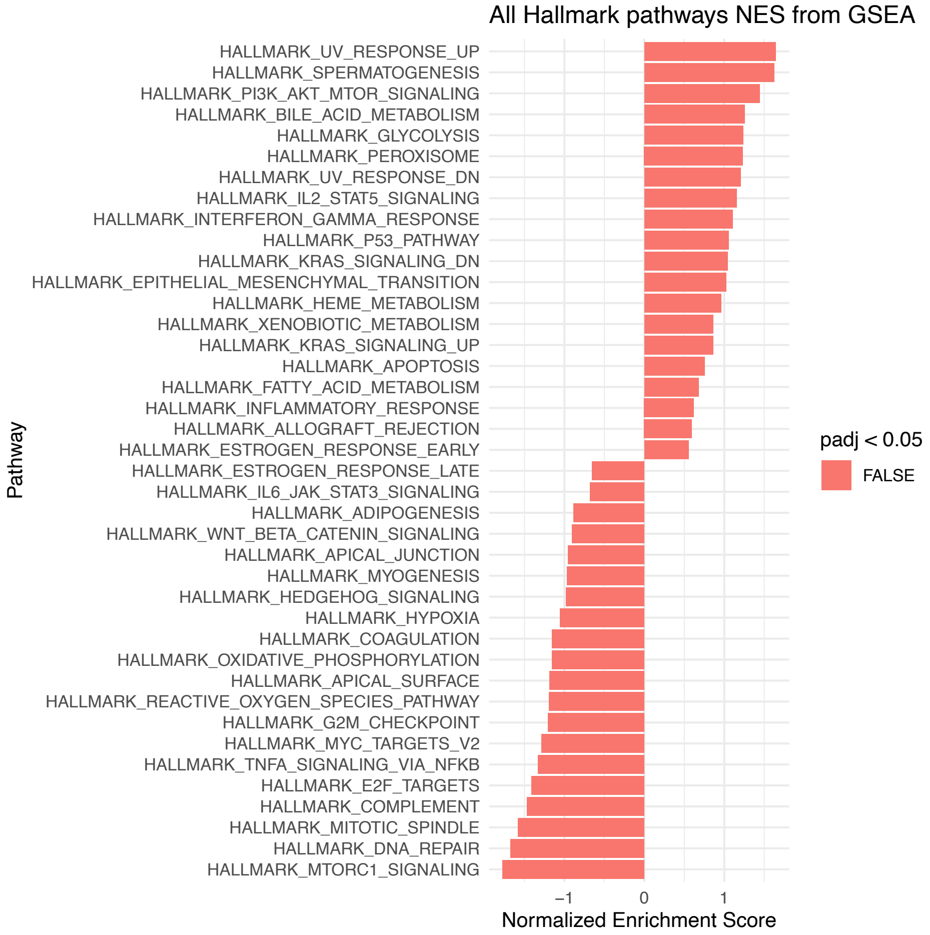

Supplementary Figure 4
Gating Strategy

Treatment: BRCA-1 null cells
TTI-621 + Niraparib

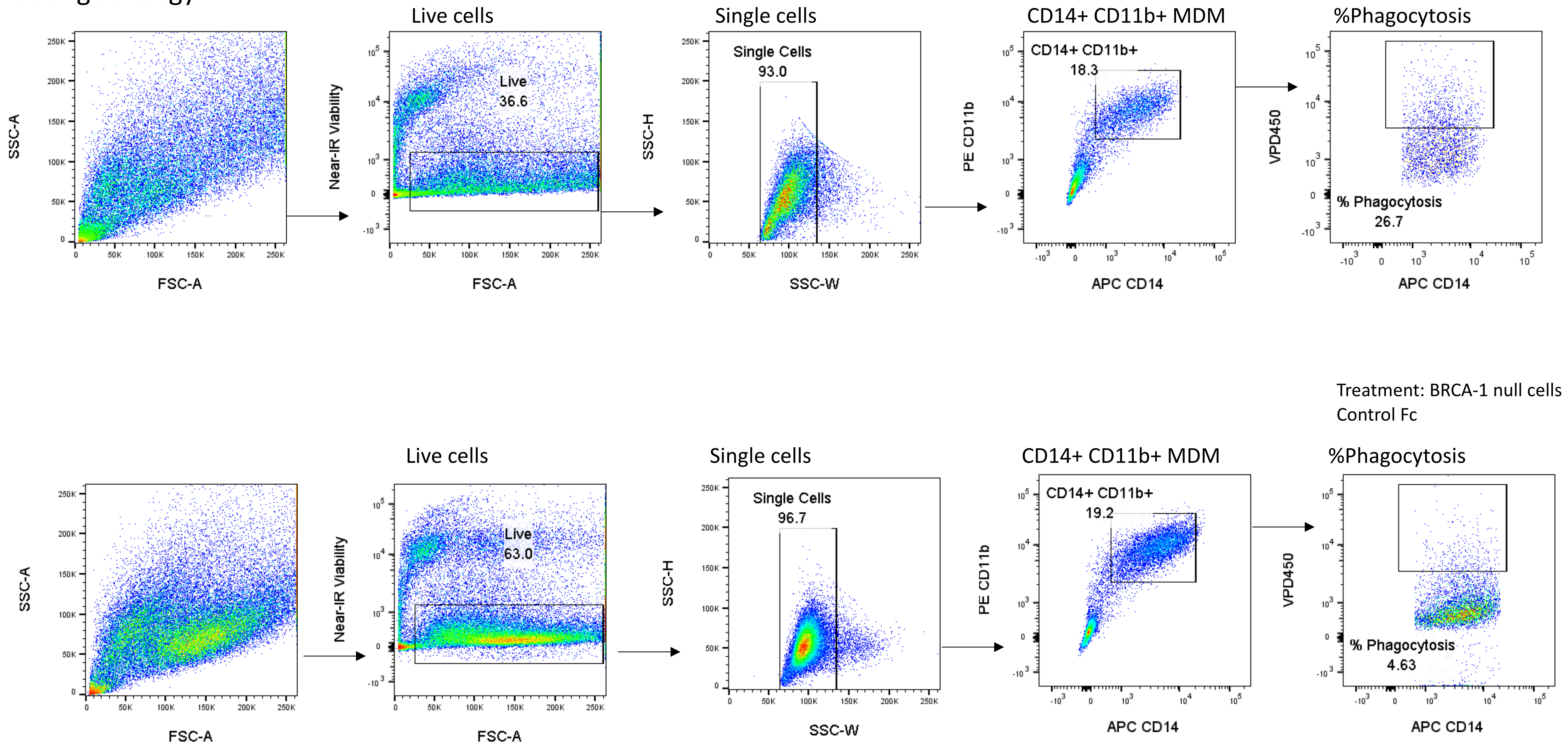

Supplementary Figure 5

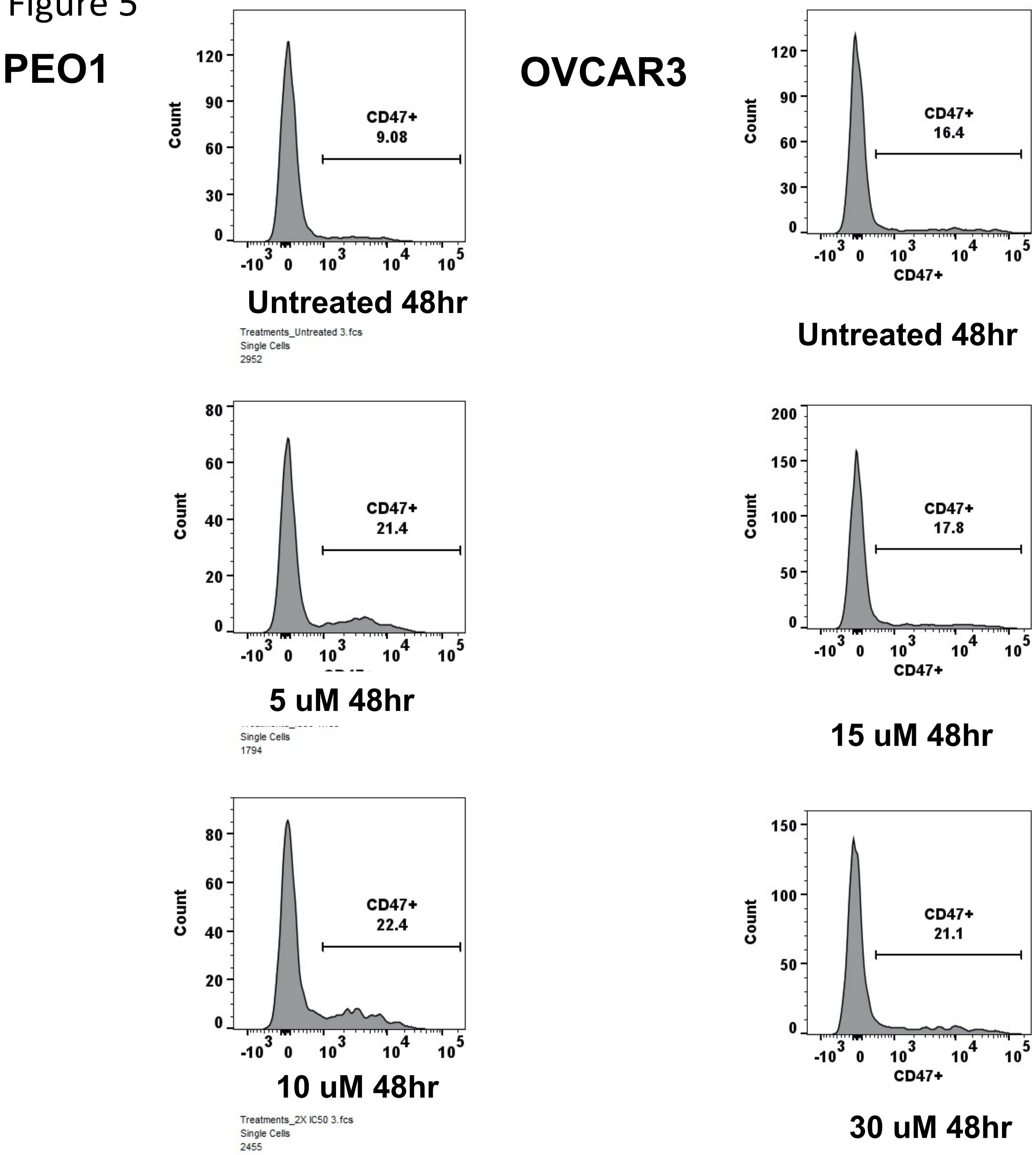

Supplementary Figure 6

A

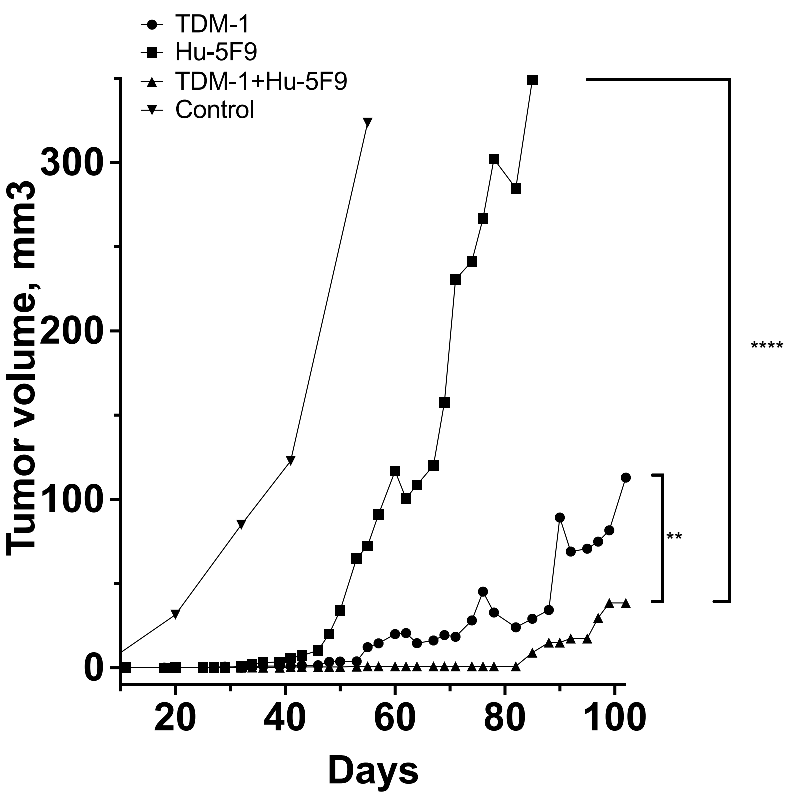

B

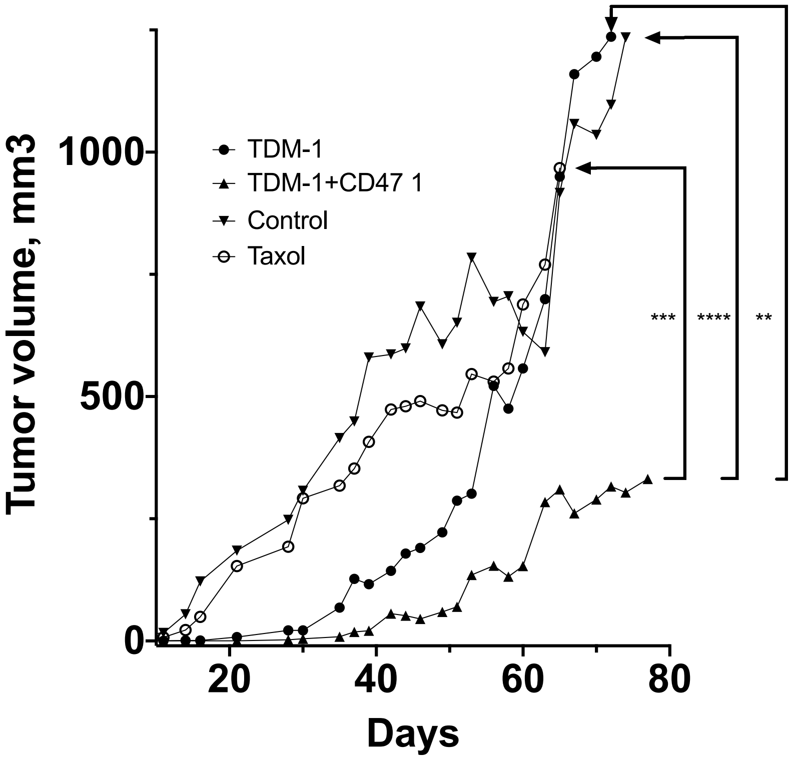

Supplement: Supplementary file 1 — Supplementary materials [file 41698_2023_418_MOESM1_ESM.pdf]
